# A Cross-Sectional Study on the Associations between Depression and Anxiety, Medication Use for These Diseases and Physical Activity Level in Spanish People with Hypertension

**DOI:** 10.3390/ijerph20031803

**Published:** 2023-01-18

**Authors:** Angel Denche-Zamorano, Belinda Basilio-Fernández, Pedro Herrera-Guerrero, Miguel Angel Garcia-Gordillo, Antonio Castillo-Paredes, Jorge Rojo-Ramos, Santiago Gómez-Paniagua, Sabina Barrios-Fernandez

**Affiliations:** 1 Promoting a Healthy Society Research Group (PHeSO), Faculty of Sport Sciences, University of Extremadura, 10003 Caceres, Spain; 2 Department of Nursing, University Center of Plasencia, University of Extremadura, 10600 Plasencia, Spain; 3 Health, Economy, Motricity and Education Research Group (HEME), Faculty of Sport Sciences, University of Extremadura, 10003 Caceres, Spain; 4 Universidad Autónoma de Chile, Talca 3467987, Chile; 5 Grupo AFySE, Investigación en Actividad Física y Salud Escolar, Escuela de Pedagogía en Educación Física, Facultad de Educación, Universidad de Las Américas, Santiago 8370040, Chile; 6 Physical Activity for Education, Performance and Health, Faculty of Sport Sciences, University of Extremadura, 10003 Caceres, Spain; 7 BioẼrgon Research Group, University of Extremadura, 10003 Caceres, Spain; 8 Occupation, Participation, Sustainability and Quality of Life (Ability Research Group), Nursing and Occupational Therapy College, University of Extremadura, 10003 Caceres, Spain

**Keywords:** physical exercise, health, hypertension, mental health

## Abstract

Hypertension (HTN) has a high prevalence in the overall population, affecting people’s mental health. Physical Activity (PA) has proven to be an effective tool to improve mental health. This study analyzed the associations between Depression and Anxiety prevalence, medication use for these disorders (antidepressants and anxiolytics) and Physical Activity Level (PAL) in people with HTN. A cross-sectional study was conducted with data from the Spanish National Health Survey 2017 (ENSE2017) with a final sample of 3228 individuals over 15 years of age with HTN who resided in Spain. Data normality was assessed through the Kolmogorov–Smirnov test. Associations between Depression and Anxiety prevalence, antidepressant and anxiolytic use and PAL were studied using a chi-square test. Possible differences between Depression and Anxiety prevalence and medication use according to the PAL were analyzed with a z-test for independent proportions. Depression or Anxiety and antidepressant and anxiolytic use odds ratios (OR) were calculated for every PAL group, taking the “Very Active” group as a reference. Risk factors were evaluated using multiple binary logistic regression. Dependency relationships were found between Depression and Anxiety prevalence, antidepressant and anxiolytic use and PAL (*p* < 0.001). The Inactive group displayed the highest prevalence and medication use according to their PAL (*p* < 0.05). Higher ORs for Depression or Anxiety and pharmacological treatments used were also found in the Inactive group compared to the other PAL groups.

## 1. Introduction

Blood pressure (BP) is caused by the blood force exerted against the vessel walls when pumped from the heart [1]. Its measures include Systolic BP (pressure in the arteries at ventricular contraction) and Diastolic BP (pressure during ventricular relaxation) which are used to determine Blood Pressure (BP), taking values below 120/80 millimetres of mercury (mmHg) as reference [2]. Hypertension (HTN) results when BP strength is too high, being a condition in which the blood vessels have persistently raised pressure, and it is diagnosed when two BP readings are equal or higher than 130–80 on two different days. BP can be classified into four groups: normal BP (SBP < 120 and DBP < 80 mmHg), high BP (SBP 120–129 and DBP < 80 mmHg), Grade 1 HTN (SBP 130–139 or DBP 80–89 mmHg) or Grade 2 HTN (SBP ≥ 140 or DBP ≥ 90 mmHg) [3,4].

HTA is a major cause of early death globally, raising the cardiac, metabolic, cerebral, renal and other organ condition risks [5,6,7]. Nowadays, more than 1 billion adults have HTN, and this figure is expected to raise to 1.5 billion in 2025. Moreover, it is the most prevalent chronic health problem in Spain, with two out of ten people diagnosed, with increasing prevalence as age increases [8]. Moreover, over half of adults with HTN are not aware of having this condition and are not managing related risk factors [9]. The modifiable risk factors are a balanced diet, limitations on alcohol and tobacco consumption, good sleep habits and performing Physical Activity (PA).

Mental health disorders are a serious global concern [10], as they can lead to worse quality of life and reduced global health [11]. Thus, depressive and anxious symptoms are associated with reduced Physical Activity Level (PAL) and self-care in individuals with mental health disorders, which are associated with increased HTN risks [8]. Moreover, Depression is underdiagnosed and under-treated when associated with chronic conditions such as HTN [12]; for example, those with cardiovascular disease have a three times greater Depression risk than those in the overall population [13,14]. HTN and depressive symptoms constitute a hazardous combination that can lead to death; thus, mental health surveillance in people with HTN is essential [15,16].

A combination of both pharmacological and non-pharmacological interventions is usually implemented in HTN and its comorbidity management [17,18]. Among the non-pharmacological approaches, healthy lifestyles and PA promotion play an important role [19]. PA is understood as any bodily movement produced by skeletal muscles with consequent energy consumption [20], and it has been considered to be an efficient indicator in physical conditions (heart disease, diabetes, stroke, cancer, obesity and HTN) [21,22,23] and mental diseases (including Anxiety and Depression) [24,25]. Hence, guidelines recommend performing 75–150 min of vigorous-intensity aerobic PA or an equivalent combination for substantial health benefits and limiting time spent in sedentary activities [26]. These recommendations apply to people with HTN [27,28], although they should be adjusted to individual needs and monitored by trained health and PA professionals [29]. Given that physical inactivity is related to higher depressive and Anxiety symptom prevalence [30] and PA is an inexpensive and easy-to-implement alternative, it is considered a suitable tool for the prevention and treatment of these diseases in individuals with HTN. PA has proved to have a positive effect on mental health through various mechanisms: through the release of endorphins, which act as natural painkillers and mood elevators; through the increase in neurotransmitter levels, such as serotonin and norepinephrine, which play a role in regulating mood and stress; and improving sleep quality and reducing stress levels, among others [31].

Spain’s policies on mental health and PA are aimed at promoting a healthy lifestyle and improving the quality of life in the population. The Spanish Ministry of Health, Consumption and Social Welfare (MSCBS) is responsible for establishing policies and programs for this purpose. Measures include creating mental health units in hospitals, promoting prevention and treatment programs for mental illness, and strengthening primary care services for mental health. In terms of PA, the government promotes programs and campaigns to encourage PA in public centres, green spaces and parks and to prevent sedentarism [32,33].

Therefore, this study aims to (1) examine the dependence relationships between Depression and Anxiety prevalence and PAL in Spanish adults with HTN; (2) evaluate possible differences in Depression and Anxiety prevalence according to PAL in Spanish adults with HTN; and (3) determine the Depression and Anxiety risks in inactives in comparison with physically active Spanish adults with HTN.

## 2. Materials and Methods

### 2.1. Design

This manuscript presents a cross-sectional study based on data from the Spanish National Health Survey 2017 (ENSE2017) [34] by using the adult questionnaire [35]. The ENSE is one of the main data collection programs performed by the MSCBS which collects data on health and health determinants in the Spanish population over 15 years of age. The ENSE is conducted by the MSCBS together with the Spanish National Institute of Statistics (INE) every five years [34]. Complete information on the objectives of the survey, its regulations, sample selection, sample calculation, data collection and processing, among other relevant aspects of its implementation can be found at https://www.sanidad.gob.es/estadEstudios/estadisticas/encuestaNacional/encuesta2017.htm (accessed on 25 August 2022).

### 2.2. Participants

The ENSE2017 uses a three-stage random stratified sampling type for participant selection including census sections (first stage), family dwellings (second stage) and individuals (third stage), employing a random sampling for each of them [34].

**Figure 1:**
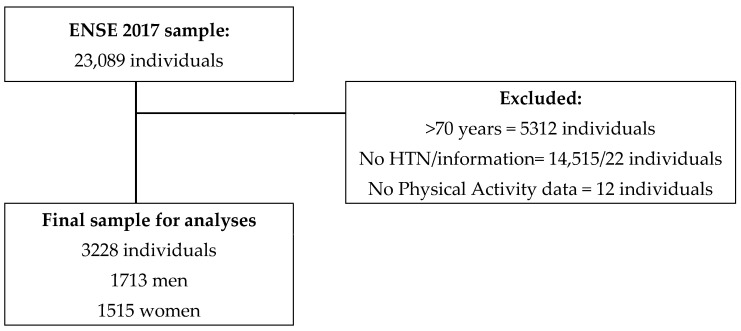
Chart outlining the study sample’s eligibility criteria.

Eligibility criteria included being younger than 70 years, having declared to have HTN (affirmative answer to item Q.25a.1 “Have you ever had hypertension?” Answers: yes, no, don’t know or no answer) and presenting data on PA-related items (Q.113–Q.117). Figure 1 describes the excluded participants with their exclusion causes and the final sample for the analysis, composed of 3228 Spanish adults with HTN who were residents in Spain and aged between 15 and 69 years.

### 2.3. Variables and Procedures

Sex: SEXOa in the ENSE2017, with options being male or female.

Age: EDADa in the ENSE2017, in years.

Depression status: item p.25a.20 in the ENSE2017, with options being “yes, not or don’t know/no answer.” For analyses involving this variable, one participant was excluded for answering “don’t know, no answer”.

Anxiety status: item p.25a.21 in the ENSE2017: with options being “yes, not or don’t know/no answer”. For analyses involving this variable, three participants were excluded for answering “don’t know, no answer”.

BMI Groups: Groupings were made according to Body Mass Index (BMI), based on responses to items p.109 (height) and p.110 (weight) in the ENSE2017. The formula BMI = Weight (in kilograms)/height^2^ (in meters) was applied, and four groups were created: <18.5, [18.5–25), [25–30) and >30. For analyses involving this variable, 138 participants were excluded for answering “don’t know, no answer” to height (82) or weight (56).

PAL Groups: Groupings were based on participants’ Physical Activity Index (PAI), derived from responses to items p.113–p.117 in the ENSE2017, which corresponded to PA carried out over the week and ranged from 0 to 67.5. Several factors were applied to the frequency, duration and intensity of participants’ PA, using an adaptation [36] to Nes et al. formula [37]. Groupings were: Inactive (PAI = 0, reported not walking one day a week, p.117), Walker (PAI = 0, reported walking at least one day a week), Active (PAI = 1–30) and Very Active (PAI < 30).

### 2.4. Statistical Analysis

IBM SPSS Statistics v. 25 software (IBM Corp., Armonk, NY, USA) was used for statistical analyses. Data normality was studied using the Kolmogorov–Smirnov test. A descriptive analysis was performed to characterise the sample, using median and interquartile range (IQR) for continuous variables (age and BMI), and absolute and relative frequencies for categorical variables (marital status, PAL, Depression status, Anxiety status, antidepressant and tranquilizer use) presented for the overall population and grouped by sex. The following non-parametrical tests were conducted: the Mann–Whitney U test to analyze possible differences between men and women in the continuous variables; a z-test for independent proportions for possible differences in proportion between sexes and the Depression status, Anxiety status, antidepressant and tranquilizer use proportions according to the PAL; and chi-square for the associations between categorical variables and sex. The contingency coefficient was calculated to assess the strength of the previous relationships interpreted according to Schubert [38]. Odds ratios for Depression and Anxiety status and antidepressant and tranquilizer use were calculated according to the PAL, using the Very Active group as a reference. Finally, multiple binary logistic regressions were performed considering as dependent variables the Depression status, Anxiety status and antidepressant and tranquilizer use, using as independent variables sex, age, PAL, BMI and civil status. For all analyses, a significance level <0.05 was considered statistically significant.

## 3. Results

**Figure 2:**
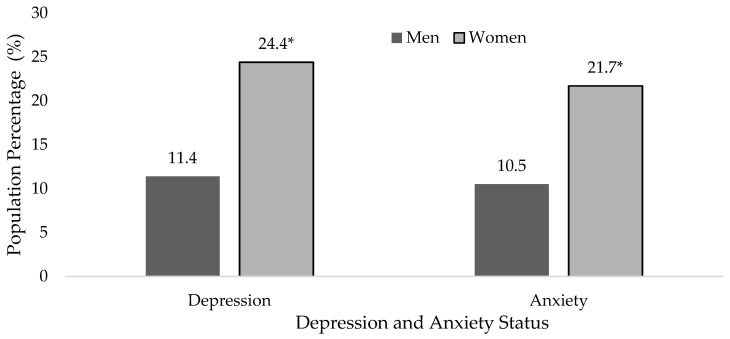
Depression and Anxiety status by sex in Spanish people with Hypertension (* *p* < 0.05 from z-test), according to the ENSE2017.

The Kolmogorov–Smirnov test found insufficient evidence to accept the normality of the data so parametric tests were used. Table S1 shows the descriptive analysis to characterise the sample according to age, BMI, marital status, PAL, Depression and Anxiety status and tranquilizer and antidepressant use, both in the overall population and divided by sex. Dependence relationships were found between sex and marital status, PAL, Depression status, Anxiety status and tranquilizer and antidepressant use (*p* < 0.001 from the chi-square test). Higher Physically Active (23.1% vs. 17.6%, *p* < 0.05 from z-test) and Very Active (8.5% vs. 5.0%, *p* < 0.05 from z-test) proportions were found in men than in women. Depression (24.4% vs. 11.4%, *p* < 0.05 from z-test) and Anxiety (21.7% vs. 10.5%, *p* < 0.05 from z-test) prevalence was also higher in women than men (Figure 2). Women also presented higher antidepressant (15.6% vs. 6.3%, *p* < 0.05 from z-test) and tranquilizer (26.6% vs. 13.7%, *p* < 0.05 from z-test) use than men.

**Figure 3:**
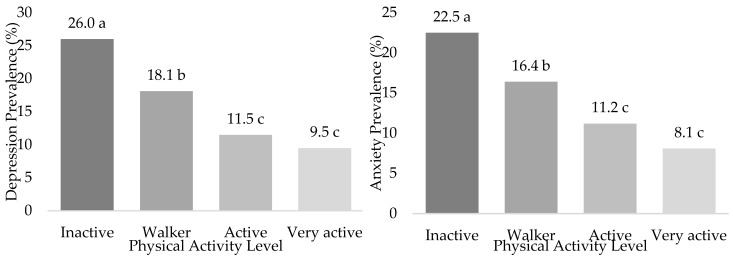
Depression and Anxiety status according to the Physical Activity Level in Spanish people with Hypertension (ENSE2017). abc (Different letters mean significant differences between Physical Activity group ratios. *p* < 0.05 from z-test).

Table S2 displays the associations between Depression and Anxiety prevalence according to the overall population, men, and women with HTN PAL. Dependency relationships were found in the overall population between the PAL and Depression and Anxiety prevalence (*p* < 0.001). The same results were found when dividing by men and women (*p* < 0.001). Depression (26.0%) and Anxiety prevalence (22.5%) was higher in the Inactive group rather than in the other PAL groups (Figure 3). This prevalence was lower as participants’ PAL increased, with significant differences between Inactives and Walkers (*p* < 0.05), as well as between these two groups and the Active and Very Active groups (*p* < 0.05), but not between the Actives and Very Actives (*p* < 0.05). The same happened when comparing by sexes.

Table 1 shows the dependence relationships found between PAL and tranquilizer and antidepressant use, both in the population with HTN and according to sex (*p* < 0.001). Inactive people had higher tranquilizer (30.9%) and antidepressant (16.7%) use compared to participants with higher PALs. In men, the group with the lowest tranquilizer (8.9%) and antidepressant use (3.3%) was the Active one; in women, this happened in the Very Active group (tranquilizers 15.8%; antidepressants 7.9%).

Table 2 shows how the Inactive and Walker groups had Depression and Anxiety risks significantly elevated when compared to the Very Active in the overall population. The Inactive ones were 3.35 times more likely to have Depression and 3.27 times more likely to have Anxiety compared to the Very Active ones. Walkers were 2.10 and 2.21 times more likely to have Depression and Anxiety, respectively, compared to the Very Active groups. Similar findings were found when divided according to sex (Table 2).

Increased risks of using tranquilizers (OR: 3.50, 95% CI: 2.22–5.51) and antidepressants (OR: 3.50, 95% CI: 1.87–6.53) were found in the Inactives compared to the Very Active group in the overall population, in men and women (Table 3).

Tables S3 and S4 show the binary multiple logistic regression models concerning Depression status, Anxiety status, and tranquilizer and antidepressant use. These models explained 9.6%, 8.0%, 8.1% and 8.7% (Nagelkerke R^2^), respectively, of the variance of the above variables. Highly active and married individuals showed lower Depression, Anxiety, and tranquilizer and antidepressant use risks.

## 4. Discussion

This study aimed to examine the associations between PAL and Anxiety and Depression status in individuals aged 15–69 years with HTN in a Spanish population cohort conducted in 2017. The key finding of this study was that physical inactivity was associated with the occurrence of anxious and depressive symptomatology. HTN is the main modifiable risk factor for cardiovascular disease and mortality worldwide, and a sedentary lifestyle is a modifiable risk factor for HTN [39]. In this condition, environmental and lifestyle factors play an important role in its onset and course [40], with PA being a protective factor in both the prevention and treatment of HTN [41], offering psychological improvements and improvements related to Depression and Anxiety, among others [42]. Patients with chronic conditions like HTN may experience many negative emotions and increased risk for mental health disorders such as Anxiety and Depression [43,44,45]. Thus, several studies conclude that people with HTN are more likely to suffer from them. One study of people with AHT is associated with psychiatric comorbidity and reduced quality of life, which is influenced by many sociodemographic and clinical factors [46]. Another study found that prehypertensive patients suffered more frequently from Depression compared with other patients with HTN, with family history, smoking and alcohol consumption being important factors [47]. Additionally, a systematic review indicates that there is evidence of a bidirectional relationship between mental health and HTN, such that elderly people with HTN would be more likely to have Depression and Anxiety, resulting in poorer quality of life and higher mortality [48].

In our study, inverse associations were found between PAL and anxious and depressive symptomatology in people with HTN, in line with findings by other authors. Physical exercise has effects on physical, mental and emotional health [49,50,51]. In the case of HTN, it is associated with a variety of improvements [52,53,54]. Depressive symptoms are common in people with HTN even in the absence of comorbidities. Thus, HTN and depressive symptoms form a combination that affects quality of life and increases all-cause mortality, so efforts to influence modifiable factors to promote suitable lifestyles should be made. Alcohol consumption and obesity predicted depressive symptoms, while non-smoking and moderate PA compared with low activity appeared to buffer depressive symptoms [55]. PA and sleep duration also impact depressive symptomatology in people with HTN [56]. Therefore, it is necessary to establish screening for Depression in patients with HTN, as untreated Depression is associated with poorer quality of life, as well as higher healthcare costs [57]. Indeed, the sense of well-being associated with aerobic exercise training may be related to the release of neurotransmitters and weight loss derived from exercise [58]. Differences were also found in people with HTN when divided by sex. One paper found significantly higher anxiety scores in women, but not in Depression [59], and another found higher Depression risks in women [55]. These results could be related to several factors, one of them being the lower PAL that women generally perform [60].

Practical applications of this study include a better understanding of relationships in the analyzed variables. As PA has proven its value as a health promotion tool, stakeholders should consider exercise benefits not only at a physical level but at a psychological one as challenges in this matter exist in people with HTN. Then, a better knowledge of these factors will help to better manage HTN psychological consequences. On the one hand, implementing PA programs to help individuals with HTN and increasing their PAL could lead to increased health, as PA reduces Anxiety and Depressive symptoms [25,30,51,61,62,63]. This, together with the fact that in the Spanish population, in line with other countries, there has been an increase in physical inactivity and sedentary habits, has led the government to promote several policies to prevent the effects of such potentially harmful habits on the population [32]. Moreover, as this study was conducted with data from the ENSE2017, the last one before the COVID-19 pandemic, it will provide a framework to compare with future health surveys. On the other hand, people with mental health issues may have greater barriers accessing or engaging PA or sports activities, therefore this must be considered when offering PA activities or programs for these populations [64,65].

This study has several limitations. On the one hand, as this is a cross-sectional study, it is impossible to analyze cause–effect relations, but only possible to identify associations. On the other hand, due to the structure of the ENSE2017, it is not possible to compare moderate and intense PA in relation to the state of mental health with the type of PA performed or to discriminate between different types or circumstances of the HTN, such as if it was well-controlled or not, as participants were not questioned about this. We also do not have information on the medication dosage, nor on other medications that could influence the mental state of the participants. Future studies should complement data collection with questionnaires and subjective perception by monitoring objective physiological data or PA dosage (duration and intensity).

## 5. Conclusions

Dependency relationships were found between Depression and Anxiety prevalence, antidepressant and anxiolytic use and PAL (*p* < 0.001). The Inactive group displayed the highest prevalence and medication use according to their PAL (*p* < 0.05). Higher ORs for Depression or Anxiety and pharmacological treatments used were also found in the Inactive group compared to the other PAL groups.

## Figures and Tables

**Table 1:** Tranquilizer and antidepressant use according to the Physical Activity Level in Spanish people with Hypertension (ENSE2017).

	Inactive		Walker		Active		Very Active					
	n	(%)	n	(%)	n	(%)	n	(%)	X^2^	df	*p*	CC
	Overall											
Tranquilizers	166a	(30.9%)	345b	(19.1%)	101bc	(15.3%)	25c	(11.3%)	60.7	3	<0.001	0.136
Antidepressants	90a	(16.7%)	203b	(11.2%)	39c	(5.9%)	12c	(5.4%)	43.5	3	<0.001	0.115
	Men											
Tranquilizers	66a	(23.9%)	120b	(13.4%)	35b	(8.9%)	13b	(9.0%)	35.1	3	<0.001	0.142
Antidepressants	32a	(11.6%)	56b	(6.3%)	13b	(3.3%)	6ab	(4.1%)	20.5	3	<0.001	0.109
	Women											
Tranquilizers	100a	(38.2%)	225b	(24.7%)	66b	(24.8%)	12b	(15.8%)	24.6	3	<0.001	0.126
Antidepressants	58a	(22.1%)	147ab	(16.1%)	26b	(9.8%)	6b	(7.9%)	18.9	3	<0.001	0.111

n (participants); % (percentages); x^2^ (Pearson’s chi-square); df (degree freedom); *p* (*p*-value from chi-square test); abc (Different letters mean significant differences between PAL group ratios). *p* < 0.05 from pairwise z-test; Tranquilizers (tranquilizer use); Antidepressants (antidepressant use); CC (contingency coefficient).

**Table 2:** Depression and Anxiety risks according to the Physical Activity Level in Spanish people with Hypertension (ENSE2017).

Physical Activity Level										
	Very Active	Active			Walker			Inactive		
Variables		OR	CI95%		OR	CI95%		OR	CI95%	
Disorder	Overall									
Depression	Ref.	1.24	0.74	2.06	2.10 *	1.32	3.35	3.35 *	2.05	5.46
Anxiety	Ref.	1.42	0.83	2.44	2.21 *	1.34	3.64	3.27 *	1.94	5.52
	Men									
Depression	Ref.	1.27	0.61	2.64	1.64	0.83	3.22	3.28 *	1.62	6.66
Anxiety	Ref.	1.11	0.51	2.42	1.82	0.90	3.68	3.18 *	1.51	6.69
	Women									
Depression	Ref.	1.11	0.54	2.27	2.00 *	1.04	3.85	2.89 *	1.45	5.75
Anxiety	Ref.	1.60	0.74	3.43	2.10 *	1.03	4.28	2.88 *	1.36	6.07

OR (odds ratio. OR > 1 Indicating a higher risk of reporting: Depression; Anxiety); CI95% (95% confidence interval of the odds ratio); * (OR with *p* < 0.05); Ref. (Reference).

**Table 3:** Antidepressant or anxiolytic use risks according to the Physical Activity Level in Spanish people with Hypertension (ENSE2017).

Physical Activity Levels										
	Very Active	Active			Walker			Inactive		
Variables		OR	CI95%		OR	CI95%		OR	CI95%	
Medication	Overall									
Antidepressants	Ref.	1.41	0.89	2.26	1.85	1.20	2.85	3.50	2.22	5.51
Anxiolytics	Ref.	1.09	0.56	2.13	2.20	1.21	4.02	3.50	1.87	6.53
	Men									
Antidepressants	Ref.	0.99	0.51	1.92	1.57	0.86	2.86	3.19	1.69	6.01
Anxiolytics	Ref.	0.79	0.29	2.11	1.54	0.65	3.65	3.04	1.24	7.45
	Women									
Antidepressants	Ref.	1.76	0.89	3.46	1.75	0.93	3.30	3.29	1.69	6.40
Anxiolytics	Ref.	1.26	0.50	3.19	2.24	0.96	5.26	3.32	1.37	8.02

OR (odds ratio. OR > 1 Indicating a higher risk to use: antidepressants; anxiolytics); CI95% (95% confidence interval of the odds ratio); Ref. (Reference).

## Data Availability

Datasets are available through the corresponding author upon reasonable request.

## Supplementary Materials

The following supporting information can be downloaded at: https://www.mdpi.com/article/10.3390/ijerph20031803/s1, Table S1: Descriptive analysis; Table S2: Relationship between Depression and Anxiety Status; and Physical Activity Level; Table S3: Logarithmic binary regression model for Depression and Anxiety risk factor; Table S4: Logarithmic binary regression model for Tranquilisers and Antidepressants Use risk factor.
