# Supplementary material for: A Cross-Sectional Study on the Associations between Depression and Anxiety, Medication Use for These Diseases and Physical Activity Level in Spanish People with Hypertension
Source: Int J Environ Res Public Health. 2023 Jan 18;20(3):1803. doi: 10.3390/ijerph20031803 (PMC9914456; doi:10.3390/ijerph20031803)
Supplement: Supplementary file 1 [file ijerph-20-01803-s001.zip › ijerph-2164503-supplementary.pdf]

Table S1. Descriptive analysis.

Variables	Men		Women		Total					
Age (Years)	n=1713		n=1515		n=3228		X²	df	p-M-W	CC
Median (IQR)	58	(13)	59	(13)	59	(14)	-	-	0.004	-
IMC (kg/m2)	n=1676		n=1435		n=3111					
Median (IQR)	28.3	(5.3)	28.0	(6.9)	28.1	(5.9)	-	-	0.001	0.001
Civil Status	n (%)		n (%)		n (%)		X²	df	p-X²	CC
Single	339	(19.8%)	204*	(13.5%)	538	(16.9%)	178.9	4	<0.001	0.229
Married	1177	(68.8%)	917*	(60.7%)	1808	(65.0%)				
Widowed	43	(2.5%)	226*	(15.0%)	661	(8.4%)				
Legally separated	45	(2.6%)	47	(3.1%)	221	(2.9%)				
Divorced	107	(6.3%)	116	(7.7%)	223	(6.9%)				
PAL										
Inactive	276	(16.1%)	262	(17.3%)	538	(16.7%)	35.2	3	<0.001	0.104
Walker	897	(52.4%)	911*	(60.1%)	1808	(56.0%)				
Active	395	(23.1%)	266*	(17.6%)	661	(20.5%)				
Very active	145	(8.5%)	76*	(5.0%)	221	(6.8%)				
Depression										
Yes	195	(11.4%)	369*	(24.4%)	564	(17.5%)	93.7	1	<0.001	0.168
No	1517	(88.6%)	1146*	(75.6%)	2663	(82.5%)				
Antidepressant										
Yes	107	(6.3%)	237*	(15.6%)	344	(10.7%)	74.5	1	<0.001	0.160
No	1605	(93.8%)	1278*	(84.4%)	2883	(89.3%)				
Anxiety										
Yes	180	(10.5%)	329*	(21.7%)	509	(15.8%)	75.9	1	<0.001	0.152
No	1531	(89.5%)	1185*	(78.3%)	2716	(84.2%)				
Tranquilisers										
Yes	234	(13.7%)	403*	(26.6%)	637	(19.7%)	85.0	1	<0.001	0.150
No	1479	(86.3%)	1112*	(73.4%)	2591	(80.3%)				

X²: Pearson's Chi-Square); df: Degree freedom; p-M-W: p-value from Mann-Whitney U test; p-X²: p-value from Chi-Square test); n: participants; %: percentage; *: Significant differents between sex ratios. p<0.05 from pairwise z-test; CC: Contingency Coefficient.

Table S2. Relationship between Depression and Anxiety Status; and Physical Activity Level.

	**Inactive**		**Walker**		**Active**		**Very active**					
	**n**	**(%)**	**n**	**(%)**	**n**	**(%)**	**n**	**(%)**	**X²**	**df**	**p**	**CC**
Overall												
Depression	140a	(26.0%)	327b	(18.1%)	76c	(11.5%)	21c	(9.5%)	53.8	3	<0.001	0.128
Anxiety	121a	(22.5%)	296b	(16.4%)	74c	(11.2%)	18c	(8.1%)	38.9	3	<0.001	0.109
Men												
Depression	54a	(19.6%)	97b	(10.8%)	34b	(8.6%)	10b	(6.9%)	24.5	3	<0.001	0.119
Anxiety	48a	(17.4%)	96b	(10.7%)	27b	(6.8%)	9b	(6.2%)	22.4	3	<0.001	0.114
Women												
Depression	86a	(32.8%)	230ab	(25.2%)	42c	(15.8%)	11bc	(14.5%)	25.2	3	<0.001	0.128
Anxiety	73a	(27.9%)	200ab	(22.0%)	47b	(17.7%)	9b	(11.8%)	12.8	3	0.005	0.109

n: participants; %: Percentages; X²: Pearson's chi-square; df: Degree freedom; p: p-value from chi-square test; abc: Different letters mean significant differents between PAL groups ratios. p<0.05 from pairwise z-test; CC: Contingency Coefficients.

Table S3. Logarithmic binary regression model for Depression and Anxiety risk factor.

**Depression**								
							**95% C.I.for EXP(B)**	
	**B**	**S.E.**	**Wald**	**df**	**Sig.**	**Exp(B)**	**Lower**	**Upper**
PAL: Very active			37.247	3	0.000			
Inactive	1.077	0.262	16.895	1	0.000	2.935	1.756	4.904
Walker	0.584	0.248	5.551	1	0.018	1.793	1.103	2.913
Active	0.183	0.269	0.463	1	0.496	1.201	0.709	2.034
Sex (Men)	0.828	0.103	64.504	1	0.000	2.288	1.870	2.800
Age (Years)	0.007	0.005	1.610	1	0.204	1.007	0.996	1.018
BMI (kg/m^2)	0.017	0.010	3.195	1	0.074	1.017	0.998	1.037
Single			47.262	4	0.000			
Married	-0.421	0.138	9.350	1	0.002	0.656	0.501	0.860
Widowed	0.170	0.198	0.738	1	0.390	1.185	0.805	1.745
Legally separated	0.145	0.294	0.244	1	0.621	1.156	0.650	2.056
Divorced	0.552	0.191	8.355	1	0.004	1.737	1.195	2.526
Constant	-3.274	0.452	52.451	1	0.000	0.038		

Anxiety								
							95% C.I.for EXP(B)	
	B	S.E.	Wald	df	Sig.	Exp(B)	Lower	Upper
PAL: Very active			29.840	3	0.000			
Inactive	1.143	0.279	16.732	1	0.000	3.136	1.814	5.424
Walker	0.746	0.265	7.933	1	0.005	2.109	1.255	3.546
Active	0.372	0.285	1.703	1	0.192	1.450	0.830	2.536
Sex (Men)	0.788	0.106	55.185	1	0.000	2.199	1.786	2.708
Age (Years)	-0.013	0.005	5.664	1	0.017	0.987	0.977	0.998
BMI (kg/m^2)	0.019	0.010	3.734	1	0.053	1.019	1.000	1.039
Single			27.344	4	0.000			
Married	-0.267	0.142	3.529	1	0.060	0.766	0.580	1.012
Widowed	0.318	0.206	2.383	1	0.123	1.375	0.918	2.059
Legally separated	0.361	0.295	1.499	1	0.221	1.435	0.805	2.560
Divorced	0.408	0.202	4.096	1	0.043	1.504	1.013	2.234
Constant	-2.550	0.453	31.639	1	0.000	0.078		

B: Understandarized beta; SE: Standard error of the regression; Wald: Wald Chi-Squared Test; Df: Degrees of freedom; Sig: Statistical significance; Exp: Exponential regression; CI: Confidence Interval; PAL: Physical Activity Level.

Table S4. Logarithmic binary regression model for Tranquilisers and Antidepressants Use risk factor.

**Tranquilisers Use**								
							**95% C.I.for EXP(B)**	
	**B**	**S.E.**	**Wald**	**df**	**Sig.**	**Exp(B)**	**Lower**	**Upper**
PAL: Very active			47.072	3	0.000			
Inactive	1.125	0.242	21.543	1	0.000	3.080	1.915	4.953
Walker	0.452	0.229	3.893	1	0.048	1.571	1.003	2.461
Active	0.266	0.246	1.169	1	0.280	1.305	0.805	2.114
Sex (Men)	0.780	0.098	63.942	1	0.000	2.181	1.802	2.640
Age (Years)	0.016	0.005	8.967	1	0.003	1.016	1.005	1.026
BMI (kg/m^2)	-0.008	0.010	0.720	1	0.396	0.992	0.974	1.011
Single			20.395	4	0.000			
Married	-0.289	0.133	4.735	1	0.030	0.749	0.577	0.972
Widowed	0.099	0.193	0.265	1	0.607	1.105	0.756	1.613
Legally separated	0.051	0.292	0.030	1	0.861	1.052	0.594	1.865
Divorced	0.331	0.192	2.959	1	0.085	1.392	0.955	2.029
Constant	-2.864	0.434	43.624	1	0.000	0.057		

Antidepressants Use								
							95% C.I.for EXP(B)	
	B	S.E.	Wald	df	Sig.	Exp(B)	Lower	Upper
PAL: Very active			30.255	3	0.000			
Inactive	1.120	0.337	11.020	1	0.001	3.065	1.582	5.937
Walker	0.632	0.323	3.825	1	0.051	1.881	0.999	3.543
Active	0.075	0.355	0.045	1	0.833	1.078	0.538	2.159
Sex (Men)	0.943	0.129	53.212	1	0.000	2.567	1.993	3.307
Age (Years)	0.012	0.007	3.431	1	0.064	1.013	0.999	1.026
BMI (kg/m2)	0.020	0.012	2.860	1	0.091	1.020	0.997	1.043
Single			17.301	4	0.002			
Married	-0.380	0.169	5.072	1	0.024	0.684	0.491	0.952
Widowed	0.094	0.234	0.160	1	0.689	1.098	0.694	1.737
Legally separated	-0.501	0.428	1.370	1	0.242	0.606	0.262	1.402
Divorced	0.271	0.236	1.318	1	0.251	1.311	0.826	2.083
Constant	-4.331	0.569	57.865	1	0.000	0.013		

B: Understandarized beta; SE: Standard error of the regression; Wald: Wald Chi-Squared Test; Df: Degrees of freedom; Sig: Statistical significance; Exp: Exponential regression; CI: Confidence Interval; PAL: Physical Activity Level.
